# The effect of exercise self-efficacy on exercise participation among Chinese university students: chain mediation of interest in sport learning and procrastination behavior

**DOI:** 10.3389/fpsyg.2025.1605854

**Published:** 2025-07-18

**Authors:** Jiang Yong, Wu Fan

**Affiliations:** School of Physical Education, Liaoning Normal University, Dalian, China

**Keywords:** exercise self-efficacy, exercise participation, interest in sports learning, procrastination behavior, chain mediation

## Abstract

**Purpose:**

To investigate the mediating role of sports learning interest and procrastination behavior between exercise self-efficacy and exercise participation among college students.

**Methods:**

A questionnaire survey was conducted with 500 Chinese university students using the Physical Activity Rating Scale (PARS-3), the Exercise Self-Efficacy Scale, the Sport Learning Interest Scale, and the Procrastination Behavior Scale.

**Results:**

Pearson correlation analysis showed that exercise self-efficacy, interest in sport learning, procrastination behavior and exercise participation were correlated with each other. The results of path analysis indicated that exercise self-efficacy was not a significant direct predictor of exercise participation. The results of mediation effects analysis indicated that sport learning interest and procrastination behavior fully mediated the relationship between exercise self-efficacy and exercise participation; the chain mediation effect of sport learning interest and procrastination behavior was significant. There were both separate mediating effects of sport learning interest and procrastination behavior between exercise self-efficacy and exercise participation, and a chain mediating effect that influenced procrastination behavior through sport learning interest, which in turn influenced exercise participation.

**Conclusion:**

Increased exercise self-efficacy promotes individual interest in sport learning, which leads to a decrease in procrastination and an increase in exercise participation.

## 1 Introduction

The World Health Organization (WHO) recommends at least 150 min of moderate-intensity exercise or 75 min of high-intensity exercise per week for adults aged 18–64 years, and some countries have incorporated this standard into the design of college and university physical education curricula. Against the background of China’s rapid educational and socio-economic development, modern college students face the double pressure of study and employment, and are able to participate in less and less time for sports, which in turn leads to the phenomenon of students’ physical health problems becoming more and more frequent, and the physical health problems appearing more and more serious. Taking the Chinese students’ physical health standard as the standard reference, more studies pointed out that college students have the lowest score in the age group, the lowest rate of attaining the standard, the highest rate of failing, and the level of physical health in the physical form dimension, the physical function dimension, the physical quality dimension all present a continuous decline in the performance of the decline of the seriousness of the overweight as well as the proportion of obese students is increasing year by year (Jie, 2019; Yun, 2018). A study shows, 63% of college students worldwide do not meet the health standard of 150 min of moderate-intensity exercise per week, and obesity rates have increased by 27% since 2000, with a 41% increase in East Asia (Phelps et al., 2024). However, the lack of sports is not confined to China, where the same emphasis on academic achievement is placed in the education system of Japan and parts of Europe, which has led to a relative weakening of physical activity in school curricula and students’ daily schedules (Mclennan and Thompson, 2015). Regular physical activity not only reduces the risk of chronic diseases such as cardiovascular disease and certain cancers and slows down the aging process (Pinckard et al., 2019), but also improves mood, helps individuals cope better with stress, and improves quality of life (Mahindru et al., 2023). For college students, active participation in physical exercise helps to cultivate a healthy body, gain psychological adjustment ability, and establish correct sports cognition and good sports behavior during college, which can lay the foundation for future sustained participation in sports (Bian, 2020).

## 2 Theoretical basis and hypothesis

### 2.1 The relationship between exercise self-efficacy and exercise participation

More studies have pointed out that exercise participation is affected by objective, subjective and environmental factors, and subjective factors are the determining factors that affect the development of exercise participation habits of college students (Gillen, 2015; Tod and Edwards, 2013; Rachel et al., 2016; Baumeister et al., 2003). Self-efficacy was proposed by the famous American psychologist Bandura (1977), refers to people’s cognitive judgments and beliefs about their ability to perform the behaviors required for the successful completion of a particular task (Bandura, 1977). In his research, Bandura emphasized that accurate judgments of self-efficacy should be made in relation to a specific domain or task, and that decontextualized measures usually have limited explanatory and predictive value (Everett et al., 2009; Pajares, 1996). Therefore, exercise self-efficacy, a variable that combines self-efficacy with sport, has more strength and depth in explaining and predicting exercise participation. Exercise self-efficacy was found to be an important factor in promoting exercise participation and sustained exercise behavior among college students (Dong et al., 2021). Some studies have proposed the role of self-efficacy in exercise behavior and found that self-efficacy is one of the key factors influencing exercise behavior. Individuals with a high sense of self-efficacy are more likely to persist in exercise and achieve good exercise results (Bill, 2023). Based on the above research, this study proposed Hypothesis H1: Exercise self-efficacy positively predicts exercise participation.

### 2.2 The mediating effect of sports learning interest

In the context of Chinese education, “sport learning” is a compulsory part of the school curriculum, encompassing such goals as skills training, character development and the transmission of traditional culture, but it has traditionally been accorded a lower status than the academic curriculum, and has often been viewed as a leisure activity rather than a serious “learning” task.

Interest in sport learning refers to a psychological disposition of learners to enjoy emotionally, develop sport cognition, and explore and sustain participation in sport (Cai et al., 2022). Individuals with a high sense of exercise self-efficacy are more likely to have positive emotional feedback and enjoyment of physical activity, which in turn enhances their behavioral motivation and interest in physical activity (Hu et al., 2016). It has been pointed out that the most important factors affecting physical activity are interest and motivation, while other factors such as policy and economy are sufficient but not necessary conditions for participation in sports, and they only limit students’ participation in sports to a certain extent (Zhang et al., 2012). Lack of interest in sport learning is an important influence on students’ boredom with physical education and low physical activity participation (Chai Jiao, 2014). It can be seen that students’ exercise self-efficacy improves at the same time, the interest in sport learning also increases accordingly, which in turn promotes the enhancement of exercise behavior (Lin and Chai, 2019). Therefore, this study proposes the hypothesis H2: interest in sport learning mediates the relationship between exercise self-efficacy and exercise participation.

### 2.3 The mediating role of procrastination behavior

Procrastination is non-essential, will cause adverse consequences of postponing behavior (Li et al., 2007; Ferrari et al., 1995), is a complex process of cognitive, emotional and behavioral (Fee and Tangney, 2000), is a self-emotional state of the management needs (Scher and Osterman, 2002). Procrastination, as a common and widespread phenomenon in college students’ daily study and life (Thøgersen-Ntoumani and Ntoumanis, 2006), can make college students lack the ability to deal with all kinds of things in their daily study and life properly, and make students themselves suffer from negative emotions (Solomon and Rothblum, 1984), and also make students’ motivation for self-determination weaker, and affect the adherence to normative behaviors (Bao and Zhang, 2006). Self-determination theory, proposed by American psychologists such as Deci and Ryan (2000), views human motivation as a dynamic continuum between external regulation and internal motivation, and categorizes motivation into internal, external, and unmotivated types based on the degree of self-integration. Individuals who are externally motivated engage in exercise participation primarily for the purpose of obtaining external rewards or avoiding internal punishments, whereas internal motivation represents a state of high autonomy and self-determination, and those who are motivated to engage in exercise participation have a deep understanding of the value of exercise participation and are actively engaged in it. Senécal et al. (1995) suggest that students who are internally motivated to learn are less likely to engage in procrastination, while those who are externally motivated to learn have a greater likelihood of procrastination (Senécal et al., 1995). Those who are externally motivated to learn have a greater likelihood of procrastination. Research has shown that procrastination and self-efficacy are common behavioral tendencies or expectations in college students’ academic life, and both have a reciprocal effect on individual behavior and motivation (Deng, 2012), and self-efficacy has a negative predictive effect on procrastination (Zou et al., 2013; Wei, 2005). At the same time,individual procrastination behavior is closely related to self-determined motivation, and college students with less procrastination behavior have a higher degree of self-determination of motivation (Xia, 2009). When individuals experience procrastination, it reduces their intrinsic motivation to participate in sports by causing time constraints or missing the optimal time to exercise, turning exercise participation into a forced or task-oriented behavior. It is inferred that there is some association between college students’ procrastination behavior and exercise participation. On this basis, the present study proposed Hypothesis H3: Procrastination behavior mediates the relationship between exercise self-efficacy and exercise participation.

### 2.4 The chain mediation effect of sports learning interest and procrastination behavior

Students who are interested in learning will show a proactive state in learning, more subjective initiative, more likely to be engaged in the learning task and feel happy, so they will not occur procrastination behavior (Mitchell, 1992). Students who are interested in learning about physical education tend to be more willing to actively participate in physical activities. In the process of participation, they can develop self-discipline and time management skills that can be transferred to other aspects of learning and life, helping them to plan their time better and complete tasks on time, thus reducing procrastination (Karaoglu and Yalçin, 2020). Meanwhile, students with higher interest in sport learning gain a sense of accomplishment through active participation in sports, which can reduce procrastination behaviors caused by anxiety or avoidance of tasks (Cao et al., 2018). In terms of sport psychology research, it has been suggested that exercise self-efficacy has a significant impact on college students’ motivation for physical activity as well as their emotional experience, which in turn determines whether or not they are able to engage in sustained learning and exercise (Li et al., 2004). Based on this, the present study proposes Hypothesis H4: There is a chain mediating role of physical education learning interest and procrastination behavior between exercise self-efficacy and exercise participation.

The innovation of this study is that the chain mediation model reveals the sequential causal relationship between variables for the first time under the same framework, and this refinement of the mediation process can help to understand the complex psychological pathways of individual exercise participation behaviors in a more comprehensive way, and provide a more detailed framework for the improvement of the relevant theoretical system. In addition, in the Chinese educational environment, “interest in sport learning” may be more influenced by collectivist culture and academic pressure, while procrastination is more significantly associated with “time management anxiety.” The chain model reveals the transmission paths of these culture-specific variables, providing empirical support for cross-cultural psychological theories in an East Asian sample. At the same time, we are trying to find a feasible way to improve the physical fitness level of college students and enhance their motivation to participate in sports.

**Figure 1:**
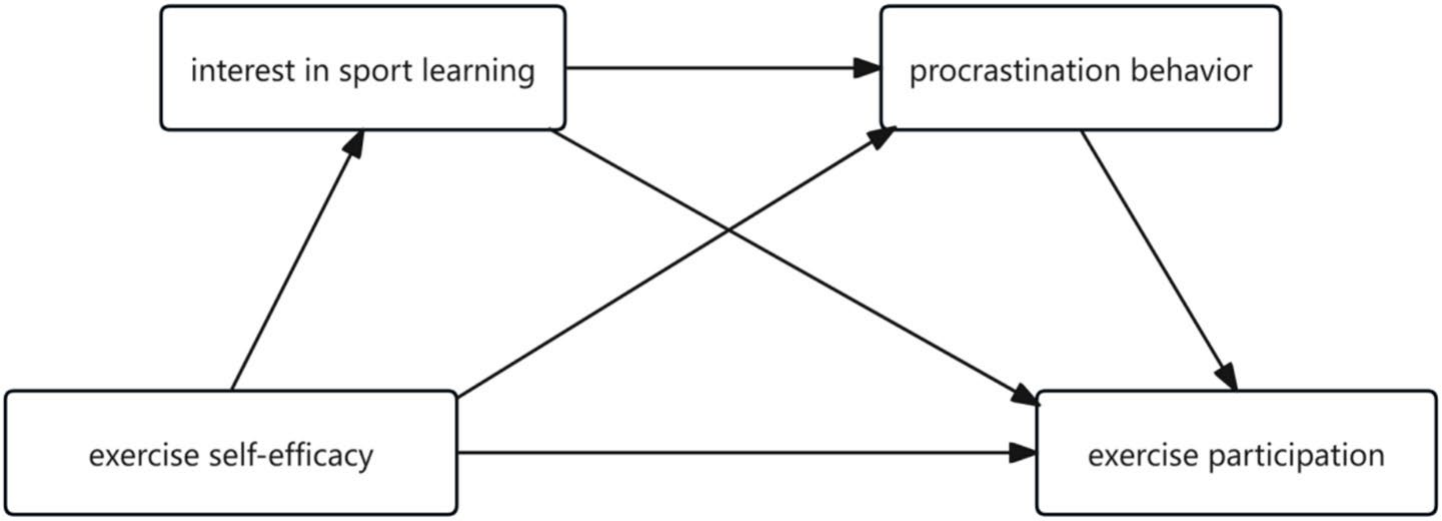
Diagram of the hypothetical model.

In summary, the hypothetical model constructed in this study is shown in Figure 1.

## 3 Research objects and methods

### 3.1 Objects of study

In order to improve the representativeness of the sample to the whole and to ensure the coverage of college student groups at different stages of study, this study used stratified random sampling based on grade level to select college students of four grades as the subjects, and distributed 500 questionnaires in total. All subjects were adults. Informed consent was obtained from the school, parents and students prior to testing. The questionnaire adhered to the principles of voluntary participation, data confidentiality, and anonymous completion. After the collection of questionnaires, the questionnaires were verified based on the principles of whether the information in the questionnaires was complete or not, and whether there was any regularity in answering the questionnaires, leaving 428 valid questionnaires, with an effective recovery rate of 85%. Among them, 231 (53.97%) were male and 197 (46.03%) were female; 154 (35.98%) were in the first year of college, 112 (26.17%) in the second year of college, 92 (21.50%) in the third year of college, and 70 (16.36%) in the fourth year of college.

### 3.2 Research tools

#### 3.2.1 Physical Activity Rating Scale (PARS-3)

The instrument used to measure exercise participation in this study was the Physical Activity Rating Scale revised by Liang (1994). The scale consists of 3 dimensions: frequency, time and intensity. For example, “What is the intensity of your physical activity?” Answer options such as: A. Light exercise (e.g., walking, doing radio gymnastics, playing goalball, etc.), B. Low intensity, less stressful exercise (e.g., recreational volleyball, table tennis, jogging, tai chi, etc.). A Likert 5-point scale was used, with frequency and intensity scored 1–5 and time scored 0–4, respectively. Exercise participation was expressed by the score of exercise volume, exercise volume = exercise intensity*(time-1)*frequency of exercise, with a score of 0–100, and a higher score indicated more exercise participation.

#### 3.2.2 Exercise Self-Efficacy Scale

The Chinese version of the Exercise Self-Efficacy Scale developed by Motl et al. (n.d.) and revised by Chen et al. (2019) was used. The scale consists of 8 questions, for example: “I can be physically active on most days of the week,” “I can be physically active even at home.” The scale is a unidimensional scale with a Likert 5-point scale with five options of strongly disagree, disagree, average, agree, and strongly agree, which are assigned a score of 1, 2, 3, 4, and 5, respectively. The total score of the scale was used to indicate the students ‘exercise self-efficacy; the higher the total score, the better the students’ exercise self-efficacy. In this study, the Cronbach’s alpha coefficient for this scale was 0.95.

#### 3.2.3 Interest in Sports Learning Evaluation Scale

The evaluation scale of college students’ interest in sports learning compiled by Gu and Xie (2012) was used, which consists of five dimensions: “postivity,” “negativity,” “skill learning,” “extracurricular activities,” and “sports concern.” The scale consists of 27 questions, such as “I would love to take physical education classes,” “Physical education is my favorite class.” The scale was scored on a 5-point Likert scale from “1″ (totally disagree) to “5″ (totally agree). The total score of the scale is used to indicate the interest of college students in physical education, and the higher the total score, the higher the individual’s interest in sport learning. The Cronbach’s alpha coefficient for this scale in this study was 0.91.

#### 3.2.4 Procrastination Behavior Scale

This study utilized the Aitken Procrastination Questionnaire, which was further revised by scholars Chen et al. (2008), and the revised scale is more in line with the situation of Chinese students. The questionnaire consists of 19 items, such as “I always wait until the last minute to get things done,” “I am often late for appointments and meetings.” The scale is based on the Likert 5-point scale, in which 9 questions such as 2, 4, 7, 11, 12, 14, 16, 17, and 18 need to be reverse scored, and the higher the total score of the scale indicates the more severe the persistent procrastination behavior in the long term. The Cronbach’s alpha coefficient for this scale in this study was 0.82.

### 3.3 Data processing

Descriptive statistics and reliability analysis of valid data were performed using SPSS 26.0. The study used SPSS 26.0 statistical software for data analysis. First, reliability was tested by Cronbach’s alpha coefficient and common method bias was detected after data collection using Harman’s one-way test. Pearson’s correlation coefficient was used to analyze the correlation between exercise self-efficacy, interest in sport learning, procrastination behavior, and exercise participation. Multiple regression analyses were conducted using Model 6 in the SPSS process macro program developed by Hayes. Bootstrap test was used to assess the significance level of mediating effects to analyze the present study.

## 4 Results and analysis

### 4.1 Common method bias test

In this study, the “This survey is for research use only” at the beginning of the questionnaire was emphasized, marked and highlighted, and the positive and negative questions were distributed in a mixed manner in order to minimize the impact of common methodological bias on the results of the study. At the same time, Harman’s one-way test was used to conduct exploratory factor analysis of all the questions included in the study variables, and there were eight factors with eigenvalues greater than 1 when the factors were not rotated, and the variance explained by the first factor was 35.28%, which was less than the critical value of 40%, indicating that there was no significant common methodological bias in the data of this study.

### 4.2 Correlation analysis of variables

Through Person correlation analysis on the data of exercise self-efficacy, exercise participation, sports learning interest and procrastination behavior of college students, it was found that exercise self-efficacy was positively correlated with exercise participation and sports learning interest, and negatively correlated with procrastination; sports learning interest was negatively correlated with procrastination and positively correlated with exercise participation; and procrastination behavior was negatively correlated with exercise participation (Table 1).

**Table 1:** Means, standard deviations and correlation coefficients of the variables (*n* = 428).

Dependent variable	M	SD	1	2	3	4
Exercising self-efficacy	3.70	0.95	1			
Interest in sport learning	3.67	0.70	0.59**	1		
Procrastination behavior	2.37	0.57	−0.40**	−0.53**	1	
Exercise participation	11.54	17.61	0.34**	0.49**	−0.36**	1

*p < 0.05; **p < 0.01; ***p < 0.001.

### 4.3 Analysis of chain mediation effects

There was a significant correlation between both exercise self-efficacy, interest in sport learning, procrastination behavior, and exercise participation, which meets the statistical requirements for further mediation effect analysis of interest in sport learning and procrastination behavior. After the above variables were jointly included in the regression equation using exercise self-efficacy as the independent variable, exercise participation as the dependent variable, and interest in sport learning and procrastination behavior as the chained mediator variables, the results of the data showed that: Gender and grade had no significant effect on interest in sport learning, procrastination behavior, and exercise participation, exercise self-efficacy had a non-significant direct predictive effect on exercise participation (*β* = 0.07, *p* > 0.05), with exercise self-efficacy being a significant positive predictor of interest in sport learning (*β* = 0.58, *p* < 0.001) and a significant negative predictor of procrastination behavior (*β* = −0.12, *p* < 0.05), while interest in sport learning was a significant negative predictor of procrastination behavior (*β* = −0.45, *p* < 0.001) and a significant positive predictor of exercise participation(*β = 0*.37, *p* < 0.001); The negative predictive effect of procrastination behavior on exercise participation was significant (*β* = −0.13, *p* < 0.05) as shown in Table 2.

**Table 2:** Regression analysis of the relationship of variables in the model.

Regression equation		Overall fit index			Significance of regression coefficients		
Outcome variable	Predictor variable	R	R^2^	F	β	SE	t
Interest in sport learning	Gender	0.59	0.34	76.62***	−0.07	0.05	−1.90
	Grade				0.03	0.02	0.77
	Exercise self-efficacy				0.58	0.03	14.79***
Procrastination behavior	Gender	0.54	0.29	44.79***	−0.07	0.05	−1.68
	Grade				−0.06	0.02	−1.38
	Exercise self-efficacy				−0.12	0.03	−2.37*
	Interest in sport learning				−0.45	0.04	−9.03***
Exercise participation	Gender	0.50	0.25	28.96***	−0.12	1.56	−0.36
	Grade				0.04	0.62	0.92
	Exercise self-efficacy				0.07	0.97	1.36
	Interest in sport learning				0.37	1.41	6.55***
	Procrastination behavior				−0.13	1.54	−2.60*

*p < 0.05; **p < 0.01; ***p < 0.001.

### 4.4 Chain mediation model test

**Figure 2:**
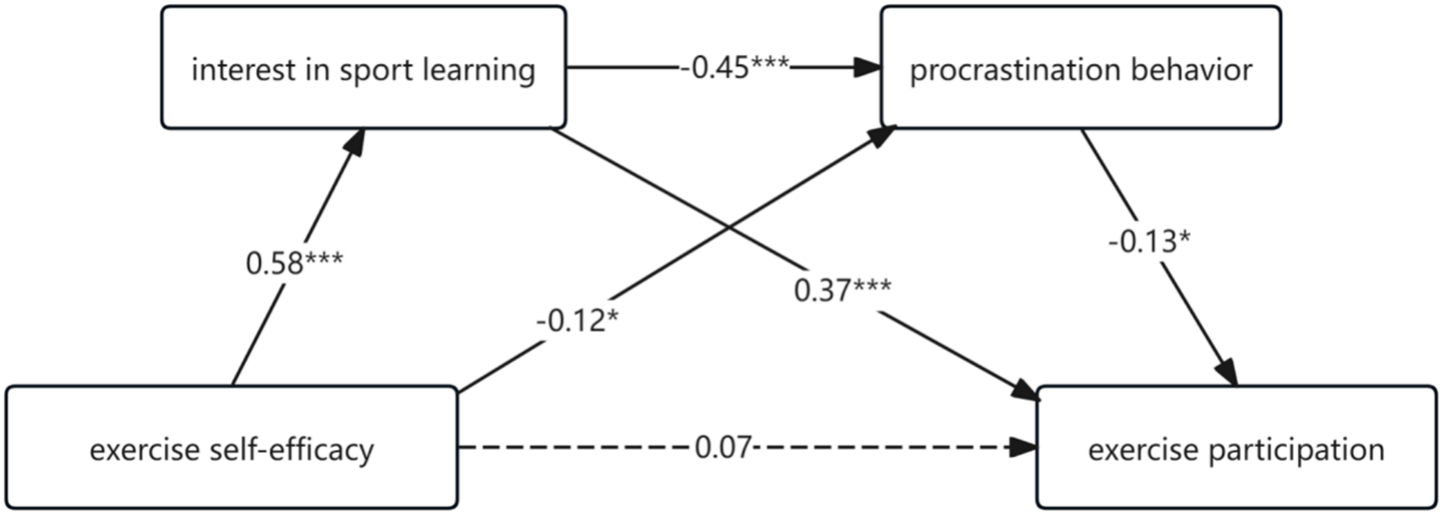
Chain-mediated model of exercise self-efficacy and exercise participation.

The relationship between exercise self-efficacy and exercise participation was further examined using the Bootstrap method of bias correction. As shown in Figure 2, the direct effect of exercise self-efficacy on exercise participation was not significant, but exercise self-efficacy acted on exercise participation by influencing interest in sport learning and procrastination behavior. This suggests that interest in sport learning and procrastination behavior play a fully mediating role in the relationship between exercise self-efficacy affecting college students’ exercise participation. The results of the mediation effect test showed that the mediation effect of sport learning interest and procrastination behavior was significant, with a total indirect effect value of 0.27. Specifically, the mediation effect of sport learning interest and procrastination behavior in the relationship between exercise self-efficacy affecting college students’ exercise participation included 3 paths (see Table 3): exercise self-efficacy →sport learning interest → exercise participation [e = 0.22, 95% CI (0.13, 0.31)], and the 95% confidence interval for the mediating effect of interest in sport learning did not include 0, indicating a significant mediating effect of interest in sport learning; second, exercise self-efficacy → procrastination behavior → exercise participation [e = 0.02, 95% CI (0.001, 0.04)], and the mediating effect of procrastination behavior was significant; and finally, exercise self-efficacy → interest in sport learning → procrastination behavior → exercise participation [e = 0.03, 95% CI (0.005, 0.07)], with a significant chain mediation effect for sport learning interest and procrastination behavior.

**Table 3:** Results of Bootstrap mediated effects analysis.

Indirect effect	Effect size	Bootstrap SE	LLCL	ULCL	Proportion of total indirect effects
Total indirect effect	0.27	0.37	0.20	0.34	100%
Ind1 Exercise self-efficacy → interest in sport learning → exercise participation	0.22	0.04	0.13	0.31	81.48%
Ind2 Exercise self-efficacy → procrastination behavior → exercise participation	0.02	0.01	0.001	0.04	7.40%
Ind3 Exercise self-efficacy →interest in sport learning → procrastination behavior →exercise participation	0.03	0.02	0.005	0.07	11.11%

## 5 Discussion

### 5.1 The effect of exercise self-efficacy on college students’ exercise participation

This study examined the relationship between exercise self-efficacy and college student athletic participation, and found that exercise self-efficacy was significantly related to college student athletic participation, but the former did not play a significant role in directly influencing the latter, which is inconsistent with the results of existing studies. The fact that this direct predictive effect was not confirmed in this study may be due to the intervention of various factors such as individual differences and external factors in the influence of exercise self-efficacy on college students’ exercise participation. Each individual has different exercise preferences, fitness levels, and interests, and these factors may influence their level of exercise participation. Bandura (1977) stated in his self-efficacy theory that individuals’ behavioral decisions are constrained by both context-specific and environmental factors (Bandura, 1977), for example, even if an individual possesses high exercise self-efficacy, his or her exercise participation may still be limited in the absence of appropriate exercise environments or social support. The direct inhibitory effect of environmental barriers on exercise participation was also illustrated in a study by Sallis et al. (2015), which indicated that the effects of exercise self-efficacy need to be translated into behavior by overcoming external barriers (Sallis et al., 2015). A study by Biddle and Mutrie (2007) noted that differences in fitness levels can lead to differences in individual tolerance to exercise intensity, which in turn affects exercise choice - students with lower fitness levels may avoid high-intensity exercise for fear of fatigue or injury, even if they have higher exercise self-efficacy (Biddle and Mutrie, 2007). A review by Hassmen et al. (2000) noted an inverted U-shaped relationship between exercise intensity and psychological distress - moderate intensity exercise enhances self-efficacy while excessive intensity may undermine confidence, which further explains the inability of exercise self-efficacy to directly predict exercise participation (Hassmen et al., 2000).

### 5.2 The mediating role of interest in sport learning

The present study found that interest in sport learning mediates the relationship between exercise self-efficacy and college students’ exercise participation, which supports hypothesis H2, which is consistent with previous studies (Hu et al., 2016; Lin and Chai, 2019) and reveals the psychological transmission law of “efficacy belief-motivation activation-behavioral participation.” From the perspective of theoretical logic, Bandura proposed self-efficacy and pointed out that the individual’s subjective judgment of ability would indirectly affect behavior through the path of “goal commitment-motivation intensity” (Chi and Xin, 2006), which is consistent with the path of “exercise self-efficacy→interest in sport learning→exercise participation” in this study. According to educational psychology, interest in learning, as the most active component of motivation (Chai Jiao, 2014), is essentially the external expression of “goal attraction” in the motivation system (Zhang, 1998). When individuals have high exercise self-efficacy, such positive beliefs are translated into interest in sport learning through the “motivational enhancement effect” described by Bandura. Self-determination theory (SDT) emphasizes that the core features of internal motivation are the “sense of interest” and “sense of autonomy” experienced by individuals in activities (Deci and Ryan, 2000), and exercise self-efficacy, as a materialization of competence beliefs, is a key factor in triggering internal motivation. Therefore, when individuals have high exercise self-efficacy, they have a natural tendency to “actively explore the enjoyment of exercise,” which is consistent with the result that “interest in sport learning positively predicts participation in exercise” in this study. In the present study, exercise self-efficacy increased the likelihood that college students would incorporate exercise participation into their behavioral plans by increasing their interest in sport learning, which is consistent with the finding that “students with higher interest in sport learning tend to view exercise as a self-selected pleasurable activity rather than as a task burden, and thus show higher frequency of participation and engagement” (Hu et al., 2016; Lin and Chai, 2019).

### 5.3 The mediating role of procrastination behavior

The results of this study showed that procrastination behavior plays a partial mediating role in the mechanism of exercise self-efficacy’s influence on college students’ exercise participation, and exercise self-efficacy can negatively influence procrastination behavior, which is consistent with previous studies (Yang, 2019), supporting hypothesis H3. Procrastination behavior is essentially a failure of self-regulation or a lack of self-control (Steel Piers, 2007). Long-term procrastination will have many negative effects on college students’ learning, physical and mental health, and will easily cause them to slacken off in physical exercise, academics and many other aspects. Some other studies have suggested that improving individual self-efficacy can improve college students’ procrastination behavior and stimulate motivation for physical activity (Wang et al., 2015). Exercise self-efficacy, as a specific manifestation of self-efficacy in sports, not only has an impact on individuals’ motivation, but also produces a positive emotional experience, which in turn increases the degree of self-determination of individuals. People with high exercise self-efficacy not only perceive pleasure in exercise, but also have a positive self-attitude that extends to other behaviors to improve procrastination (Li et al., 2022). As procrastination decreases, exercise procrastination also improves, which leads to increased participation.

### 5.4 Chain mediating role of sport learning interest and procrastination behavior

The results of this study show that the effects of exercise self-efficacy on college students’ exercise participation are not only realized through the separate mediating paths of sport learning interest and procrastination behavior, but also through the chain mediating effect of the two. In terms of theoretical mechanisms, the enhancement of exercise self-efficacy can enhance an individual’s interest in sport learning through the affective activation pathway. Bandura (1977) self-efficacy theory proposes that high self-efficacy individuals are more inclined to actively engage in challenging activities and experience positive emotions. The present study further found that interest in sport learning, as an affective mediating variable, is essentially an individual’s cognitive preference and emotional engagement in physical education learning (Hagger and Chatzisarantis, 2014). When interest in sport learning is heightened, individuals’ intrinsic motivation for sport activities increases, thereby inhibiting procrastination behaviors that conflict with exercise participation. This finding is consistent with Deci and Ryan (2000) self-determination theory, and the activation of internal motivation reduces barriers to the execution of goal behaviors.

At the level of behavioral transmission, exercise self-efficacy indirectly influences exercise participation through behavioral regulation pathways. Research has shown that people with high exercise self-efficacy are better at managing daily behaviors through strategies such as goal setting and self-monitoring (Schwarzer, 2014). This study suggests that increased interest in sport learning can reduce the tendency to procrastinate, resulting in a chain effect of “emotion-driven-behavior-optimized.” This mechanism can be explained within the framework of Baumeister’s ego depletion theory, which states that positive affective experiences complement self-control resources, thereby weakening the hindrance of procrastination behaviors to exercise participation (Baumeister and Vohs, 2007). The present study proposes chain mediation as an integration of research on the relationship between physical education learning interest and procrastination behavior and exercise participation, which is conducive to a more comprehensive understanding of the internal mechanism of action of exercise self-efficacy on the impact of college students’ exercise participation, and is of guiding significance for the comprehensive development of college physical education as well as the cultivation of college students’ athletic ability and their physical and mental health development.

## 6 Conclusions and recommendations

### 6.1 Conclusion

Exercise self-efficacy, interest in sport learning, and procrastination behaviors are bipartisanly related to college student exercise participation.

Sports learning interest, procrastination behavior in the exercise self-efficacy on college students’ exercise participation in the process of the influence of not only a simple intermediary role, but also through the sports learning interest and procrastination behavior chained to the college students’ exercise participation, this indirect mediation effect of the students’ physical and mental qualities of the healthy development of an important impact.

### 6.2 Recommendations

The university stage, as a key transition period of individual growth, plays a decisive role in shaping worldview, outlook on life and values (Astin, 1993). During this period, cultivating students’ exercise participation habits is not only about physical health, but also an important way to promote psychological health and academic achievement (Doré et al., 2018). Regular physical activity can significantly improve the mental toughness, emotional stability and cognitive function of college students, thus laying the foundation for their lifelong health (Ratey and Spark, 2008). It is important to note that, as mentioned above, there are also cases in Japan and parts of Europe where physical education is weakened due to educational pressures. Compared with these countries, although there are differences in cultural backgrounds and education systems, the impact of common academic pressure on students’ sports participation is similar. Future research can further compare the similarities and differences between different countries on this issue, and explore more universal intervention strategies to improve the sports participation of college students in different cultural backgrounds.

Schools, teachers and parents need to build a collaborative cultivation mechanism to systematically enhance students’ interest in sports and exercise self-efficacy. Schools should optimize the physical education curriculum system, incorporate emerging sports, and build a participation ecosystem based on club activities and campus sports and cultural festivals; teachers need to innovate teaching strategies, strengthen students’ success experience through stepped goal setting and specific feedback, and stimulate intrinsic motivation through game-based teaching and differentiated instruction; and parents need to establish a family sports support system to create an atmosphere for sports. By activating the internal drive of interest through diversified exercise experiences and enhancing self-efficacy through continuous successful experiences, a virtuous cycle of “interest-driven participation - efficacy reinforcing persistence” is formed, which ultimately promotes the synergistic development of students’ physical functions and psychological qualities, and realizes the deep transformation from exercise participation to physical and mental health.

### 6.3 Research shortcomings and prospects

First of all, this study is a cross-sectional survey study, and although it was conducted based on theoretical foundations and the results of previous studies, it is still unable to make exact causal inferences. It is suggested that subsequent researchers can utilize a longitudinal research design and adopt a cross-lagged correlation design to examine the process mechanism of exercise self-efficacy’s influence on college students’ exercise participation. Second, this study only examined the chain-mediated effects of interest in sports learning and procrastination behavior, and it is recommended that subsequent researchers continue to examine personality factors, lifestyle, and other variables that are closely related to college students’ exercise participation. Finally, the population of this study was college students, and the variables studied may have similar relationships in other groups, so it is recommended that future research may consider expanding the scope of the study population.

## Data Availability

The original contributions presented in the study are included in the article/supplementary material, further inquiries can be directed to the corresponding author/s.
